# Different characteristics of bloodstream infection during venoarterial and venovenous extracorporeal membrane oxygenation in adult patients

**DOI:** 10.1038/s41598-021-89108-4

**Published:** 2021-05-04

**Authors:** Hyoung Soo Kim, Sunghoon Park, Ho Hyun Ko, Sang Ook Ha, Sun Hee Lee, Yong Kyun Kim

**Affiliations:** 1 Department of Thoracic and Cardiovascular Surgery, Hallym University Sacred Heart Hospital, Hallym University College of Medicine, Anyang, Korea; 2 Division of Pulmonary, Allergy and Critical Care Medicine, Department of Internal Medicine, Hallym University Sacred Heart Hospital, Hallym University College of Medicine, Anyang, Korea; 3 Department of Emergency Medicine, Hallym University Sacred Heart Hospital, Hallym University College of Medicine, Anyang, Korea; 4 Division of Infectious Diseases, Department of Internal Medicine, Hallym University Sacred Heart Hospital, Hallym University College of Medicine, Anyang, Korea

**Keywords:** Infectious diseases, Microbiology, Diseases

## Abstract

Currently, there is scarcity of data on whether differences exist in clinical characteristics and outcomes of bloodstream infection (BSI) between venoarterial (VA) and venovenous (VV) extracorporeal membrane oxygenation (ECMO) and whether they differ between *Candida* BSI and bacteremia in adult ECMO patients. We retrospectively reviewed data of patients who required ECMO for > 48 h and had BSIs while receiving ECMO between January 2015 and June 2020. Cases with a positive blood culture result within 24 h of ECMO implantation were excluded. We identified 94 (from 64 of 194 patients) and 38 (from 17 of 56 patients) BSI episodes under VA and VV ECMO, respectively. Fifty nine BSIs of VA ECMO (59/94, 62.8%) occurred in the first 2 weeks after ECMO implantation, whereas 24 BSIs of VV ECMO (24/38, 63.2%) occurred after 3 weeks of ECMO implantation. Gram-negative bacteremia (39/59, 66.1%) and gram-positive bacteremia (10/24, 41.7%) were the most commonly identified BSI types in the first 2 weeks after VA ECMO implantation and after 3 weeks of VV implantation, respectively. Timing of *Candida* BSI was early (6/11, 54.5% during the first 2 weeks) in VA ECMO and late (6/9, 66.7% after 3 weeks of initiation) in VV ECMO. Compared with bacteremia, *Candida* BSI showed no differences in clinical characteristics and outcomes during VA and VV ECMO, except the significant association with prior exposure to carbapenem in VA ECMO (vs. gram-negative bacteremia [*P* = 0.006], vs. gram-positive bacteremia [*P* = 0.03]). Our results suggest that ECMO modes may affect BSI clinical features and timing. In particular, *Candida* BSI occurrence during the early course of VA ECMO is not uncommon, especially in patients with prior carbapenem exposure; however, it usually occurs during the prolonged course of VV ECMO. Consequently, routine blood culture surveillance and empiric antifungal therapy might be warranted in targeted populations of adult ECMO patients, regardless of levels of inflammatory markers and severity scores.

Bloodstream infection (BSI) is a well-known infectious complication of extracorporeal membrane oxygenation (ECMO) with the prevalence ranging from 3 to 18% in adult patients, and may be associated with organ failure^1,2^. The multiple predisposing factors and potential poor outcomes of BSI in patients requiring ECMO highlights the need for better understanding of the epidemiology (including the clinical manifestations) of BSI, a condition caused by various infectious agents^1^. However, best practice is difficult to establish because previous studies regarding BSI during ECMO therapy included heterogenous study populations with differences in characteristics between the venoarterial (VA) ECMO and venovenous (VV) ECMO groups and with a small number of patients only^2–6^.

In several studies on nosocomial infections, for BSI under ECMO, the outcomes may differ depending on the causative pathogens, and some organisms were identified as risk factors for increased mortality^3,7,8^. For appropriate antimicrobial prophylaxis and optimal management of BSI and to reduce mortality, it is important to know the epidemiology of BSI. *Candida* BSI has been reported to account on average for 13% of BSIs during ECMO^1,9^, and it is associated with increased mortality^10,11^. Although a few recent studies evaluated nosocomial infections that included *Candida* BSI in a homogenous group of adult patients under VA and VV ECMO^12–15^, there have been no comparative clinical studies on the characteristics of patients receiving VA and VV ECMO. In addition, there is paucity of data regarding the clinical characteristics and outcomes of *Candida* BSI as distinct from those of bacteremia, in adult patients under ECMO.

Therefore, in this study, we aimed to evaluate whether the clinical characteristics and outcomes of BSI are different between VA and VV ECMO and whether these differ between *Candida* BSI and bacteremia in adult patient groups of VA and VV ECMO.

## Methods

### Study design and population

This retrospective study was conducted at Hallym University Sacred Heart Hospital, an 829-bed university-affiliated hospital in Anyang, Korea. We reviewed the medical charts of the 272 adult patients over the age of 18 years in the surgical intensive care units (SICU) who received VA ECMO between January 2015 and June 2020, and excluded 78 patients because they received ECMO for less than 48 h. In addition, we reviewed 61 patients over the age of 18 years in SICU who received VV ECMO, and excluded 5 patients with less than 48 h of ECMO.

BSI cases were included if patients had a positive blood culture result after 24 h of ECMO initiation but within 48 h of ECMO discontinuation, and they should have been under ECMO for more than 48 h. A subsequent positive blood culture result showing the same organism and antibiotic susceptibility pattern within 7 days was classified as 1 BSI case. BSI cases were excluded if a positive blood culture result was obtained within 24 h of ECMO initiation because such cases were not clearly associated with ECMO. We included at least one positive blood culture each for gram-negative organism, *Candida* species, and *Staphylococcus aureus*. In addition, at least one positive blood culture for *Enterococcus* without other identifiable secondary source of infection was included. The common gram-positive skin contaminants, such as coagulase-negative *Staphylococci* and *Corynebacterium* species, were not included because they did not account for high morbidity or mortality in patients, and all blood samples were drawn from indwelling catheters in the present study. The study was approved by the Hallym University Sacred Heart Hospital Institutional Review Board (IRB), which waived the requirement for informed consent owing to the retrospective design of the study. All methods were carried out in accordance with relevant guidelines and regulations.

### Data collection and definitions

Clinical data regarding patients’ epidemiological and clinical characteristics as well as outcomes were collected.

Severity was stratified according to the sequential organ failure assessment (SOFA) score at the time of BSI^16^, Simplified Acute Physiology Score (SAPS) II at the time of BSI^17^, and SAPS III at ICU admission^18^. An immunocompromised state was defined as (1) hematologic malignancy, (2) active solid tumor with specific antitumor treatment within the last 1 year, (3) solid-organ transplant, (4) long-term corticosteroid use (> 7.5 mg of prednisone or equivalent per day for more than 3 months), or (5) high-dose corticosteroid use (> 1 mg/kg of prednisone or equivalent for more than 1 week within the last 3 months)^19^. Data regarding the use of continuous renal replacement therapy (CRRT) as well as other life-support procedures, including receipt of total parenteral nutrition (TPN), and systemic antibiotic use (> 48 h administration) within 14 days prior to BSI were collected. Other baseline characteristics including demographics (age, gender), ICU days before ECMO implantation, days between ECMO implantation and BSI, duration of ECMO support, levels of inflammatory markers (lactate, C-reactive protein, procalcitonin) on the day of BSI diagnosis were collected. Outcomes, including the success of ECMO weaning (weaning followed by stable survival for more than 48 h) and survival to discharge (in-hospital mortality), were also evaluated.

The prevalence of BSI during ECMO was expressed as the number of infected patients to the overall number of ECMO patients (%), and incidence of BSI during ECMO was expressed as the number of infectious episodes to the overall duration of the ECMO course (cases per 1,000 ECMO days).

### Procedures and management of ECMO patients

The procedures and management of ECMO patients are as described in several recent studies, by our institution^20–22^. VA ECMO was indicated for acute circulatory failure with (1) systolic blood pressure < 80 mmHg despite adequate intravascular volume replacement and high-dose vasopressor infusion (norepinephrine > 0.5 μg/kg/min) or (2) cardiac arrest that lasted for > 10 min despite cardiopulmonary resuscitation. VV ECMO was indicated for acute respiratory failure with a ratio of arterial oxygen tension to inspired oxygen fraction (PaO_2_/FiO_2_) < 100 on ventilation with FiO_2_ of 100% and low-dose vasopressor infusion (norepinephrine < 0.5 μg/kg/min). Patients were excluded if they had ongoing intracranial hemorrhage, terminal malignancy, loss of ability to independently perform their activities of daily living, or unwitnessed cardiac arrest. As for site of ECMO cannulation, all patients received extrathoracic ECMO using femoral artery and vein.

Strategies regarding antimicrobial prophylaxis, blood culture surveillance, and infection prevention practices are as follows. In VA ECMO, we routinely administer a first-generation cephalosporin as antibacterial prophylaxis for the first 48 h at the initiation of ECMO, and piperacillin–tazobactam for initial empiric coverage in cases of suspected sepsis. In VV ECMO, all of patients would have already been administered empiric antibiotics at ECMO initiation. Despite the lack of a standardized protocol for antibiotic regimen, the combination regimen of piperacillin–tazobactam and levofloxacin is for the initial empiric coverage in most cases, and initially prescribed antibiotics do not include colistin and meropenem. Routine antifungal prophylaxis is not prescribed for both VA and VV ECMO, and specific antibiotic regimens, according to the patients’ clinical situation and culture results, are discussed with the physicians in the infectious diseases division. For all study patients, daily blood culture surveillance, sampled from the central venous catheter and arterial catheter, was performed from the day of ECMO implantation throughout the course of ECMO therapy. Infection prevention practices include hand hygiene with an alcohol-based sanitizer, daily observation of catheter insertion sites, and disinfection with chlorhexidine solution followed by weekly change of chlorhexidine-impregnated transparent dressing or daily change of sterile gauze dressing at catheter insertion sites.

### Statistical analyses

Categorical variables were compared using the *χ*^2^ or Fisher’s exact test, and continuous variables were compared using the Mann–Whitney *U* test, as appropriate. Logistic regression analysis was used to compare variables associated with BSIs in ECMO patients. All tests of significance were two-tailed, and a *P* value < 0.05 was considered to indicate statistical significance. All statistical analyses were performed using IBM SPSS Statistics for Windows, version 21 (IBM Corp., Armonk, N.Y., USA).

## Results

### Patients and microbiological characteristics

During the study period, we identified 194 and 56 patients who received VA and VV ECMO, respectively. Of these, 94 episodes of BSI in 64 patients under VA ECMO and 38 episodes of BSI in 17 patients under VV ECMO were confirmed. We excluded 9 (7 coagulase-negative *Staphylococci* and 2 *Corynebacterium* species) and 4 (4 coagulase-negative *Staphylococci*) blood cultures of the common gram-positive skin contaminants in VA and VV ECMO patients, respectively.

The causative organisms of BSI are detailed in Table 1. In those who received VA ECMO and VV ECMO, gram-negative bacteremia was the most common cause of BSI (58/94, 61.7% vs.16/38, 42.1%), followed by gram-positive bacteremia (25/94, 26.6% vs. 13/38, 34.2%) and *Candida* BSI (11/94, 11.7% vs. 9/38, 23.7%). *Acinetobacter baumannii* (*A. baumannii*) (36/94, 38.3%) and *Enterococcus faecium* (*E. faecium*) (9/38, 23.7%) were the most frequently isolated pathogens in VA and VV ECMO, respectively. Eleven cases of *Candida* BSI in VA ECMO and nine cases of *Candida* BSI in VV ECMO were also identified. *Candida albicans* (4/11, 36.4%) and *Candida glabrata* (4/9, 44.4%) were the most frequently cultured organisms in VA and VV ECMO, respectively.

**Table 1:** Pathogens associated with 132 bloodstream infections in 81 extracorporeal membrane oxygenation (ECMO) patients.

Mode of ECMO	Types of pathogens		
	Fungi (20 isolates)	Gram-negative pathogens (74 isolates)	Gram-positive pathogens (38 isolates)
Venoarterial (94 bloodstream infection [BSI] episodes)	*Candida albicans* 4	*Acinetobacter baumannii* 36	*Staphylococcus aureus* 13
	*Candida glabrata* 3	*Klebsiella pneumoniae* 11	*Enterococcus faecium* 10
	*Candida parapsilosis* 2	*Pseudomonas aeruginosa* 4	*Enterococcus faecalis* 2
	*Candida tropicalis* 1	*Enterobacter aerogenes* 3	
	*Candida famata* 1	*Serratia marcescens* 2	
		*Escherichia coli* 1	
		*Proteus mirabilis* 1	
Venovenous	*Candida glabrata* 4	*Acinetobacter baumannii* 8	*Enterococcus faecium* 9
(38 BSI episodes)	*Candida tropicalis* 3	*Klebsiella pneumoniae* 2	*Staphylococcus aureus* 4
	*Candida albicans* 1	*Serratia marcescens* 2	
	*Candida parapsilosis* 1	*Enterobacter cloacae* 2	
		*Pseudomonas aeruginosa* 1	
		*Morganella morganii* 1	

### Comparisons of the characteristics and outcomes of BSIs under VA and VV ECMO

Table 2 shows the differences in clinical and microbiological features as well as outcomes of BSIs under either VA or VV ECMO.

**Table 2:** Clinical characteristics and outcomes of the 132 bloodstream infections during venoarterial (VA) and venovenous (VV) extracorporeal membrane oxygenation (ECMO).

Variables	Venoarterial (n = 94)	Venovenous (n = 38)	*P* value*
**Age, median years (IQR)**	59 (46–68)	57 (50–67)	0.30
**Male**	69 (73)	27 (71)	0.78
**Candida BSI**	11 (12)	9 (24)	0.08
**Gram-positive bacteremia**	25 (27)	13 (34)	0.38
**Gram-negative bacteremia**	58 (61)	16 (42)	**0.04**
Carbapenem-resistant pathogen^a^	46 (49)	14 (37)	0.21
**ICU days before ECMO implantation, median (IQR)**	1 (0–2)	3 (1–10)	** < 0.001**
**Time between ECMO implantation and BSI, median days (IQR)**	13 (7–19)	28 (14–53)	** < 0.001**
**Duration of ECMO, median days (IQR)**	17 (13–25)	48 (31–85)	** < 0.001**
**Immunocompromised state**	55 (59)	30 (79)	**0.026**
Hematological malignancy	2 (2)	0 (0)	> 0.99
Active solid tumor^b^	1 (1)	1 (3)	0.49
Solid-organ transplant	5 (5)	11 (29)	** < 0.001**
Long-term corticosteroid use^c^	1 (1)	2 (5)	0.20
High-dose corticosteroid use^d^	52 (55)	21 (55)	0.99
**SAPS III at ICU admission**	52 (41–64)	41 (26–57)	** < 0.001**
**SOFA at the time of BSI**	12 (10–14)	10 (7–14)	** < 0.001**
**SAPS II at the time of BSI**	63 (51–76)	48 (40–53)	** < 0.001**
**Inflammatory markers on the day of BSI**^e^			
Lactate, mmol/L	4.5 (2.8–8.8)	3.1 (2.5–4.3)	**0.002**
CRP, mg/dL	13.4 (6.3–27.2)	16.6 (9.9–22.1)	0.28
Procalcitonin, ng/mL	4.5 (1.6–15.3)	1.5 (0.8–5.9)	**0.002**
**CRRT prior to BSI**^f^	73 (78)	29 (76)	0.87
**TPN prior to BSI**^f^	56 (60)	25 (66)	0.51
**Outcomes**			
ECMO weaning	26 (28)	11 (29)	0.88
Survival to discharge^g^	13/64 (20)	5/17 (29)	0.42

BSI, bloodstream infection; CRP, C-reactive protein; CRRT, continuous renal replacement therapy; ICU, intensive care unit; IQR, interquartile range; SAPS, Simplified Acute Physiology Score; SOFA, sequential organ failure assessment; TPN, total parenteral nutrition.

*P values with significant difference are indicated in **bold.**

Data are presented as No. (%) unless indicated otherwise.

^a^ Resistant to at least one of the carbapenem antibiotics (ertapenem, meropenem, or imipenem).

^b^ Antitumor treatment within the previous year.

^c^ > 7.5 mg of prednisolone or equivalent per day for more than 3 months.

^d^ > 1 mg/kg of prednisolone or equivalent per day for more than 1 week within the last 3 months.

^e^The worst value was recorded, if more than 1 value were checked in the same day.

^f^ > 48 h administration within 14 days prior to BSI.

^g^Overall survival rates evaluated in patient-based, not episode-based (94 episodes of BSI in 64 patients under VA ECMO and 38 episodes of BSI in 17 patients under VV ECMO were confirmed in the present study).

Of the clinical and microbiological features, as well as outcomes of BSI, significant differences were present between those who received VA ECMO and VV ECMO with gram-negative bacteremia (58/94, 61.7% vs. 16/38, 42.1%, *P* = 0.04). Although *Candid*a BSI was more frequent in VV ECMO, no difference was found between VA ECMO and VV ECMO. BSI episodes of VA ECMO had significantly shorter ICU days before ECMO implantation (median, interquartile range [IQR]; 1 [0–2] vs. 3 [1–10] days; *P* < 0.001), days between ECMO implantation and BSI (median [IQR]; 13 [7–19] vs. 28 [14–53] days; *P* < 0.001), and duration of ECMO (median [IQR]; 17 [13–25] vs. 48 [31–85] days; *P* < 0.001) than those of VV ECMO. The severity scores, including SAPS III at ICU admission (median [IQR]; 52, [41–64] vs. 41 [26–57]; *P* < 0.001), SOFA scores at the time of BSI (median [IQR]; 12 [10–14] vs. 10 [7–14]; *P* < 0.001); and SAPS II at the time of BSI (median [IQR]; 63 [51–76] vs. 48 [40–53]; *P* < 0.001) were significantly higher in VA ECMO than in VV ECMO. In addition, levels of inflammatory markers such as lactate (median [IQR]; 4.5 [2.8–8.8] vs. 3.1 [2.5–4.3] mmol/L; *P* = 0.002) and procalcitonin (median [IQR]; 4.5[1.6–15.3] vs. 1.5 [0.8–5.9] ng/mL; *P* = 0.002) at the time of BSI were significantly higher in VA ECMO than in VV ECMO. Overall survival rates were 20.3% (13/64) and 29.4% (5/17) in VA and VV ECMO patients with BSI, respectively, which tended to be lower than patients without BSI (57.7% (75/130) in VA ECMO and 69.2% (27/39) in VV ECMO). However, there were no significant differences in outcomes such as ECMO weaning and survival to discharge between BSI during VA and VV ECMO.

**Figure 1:**
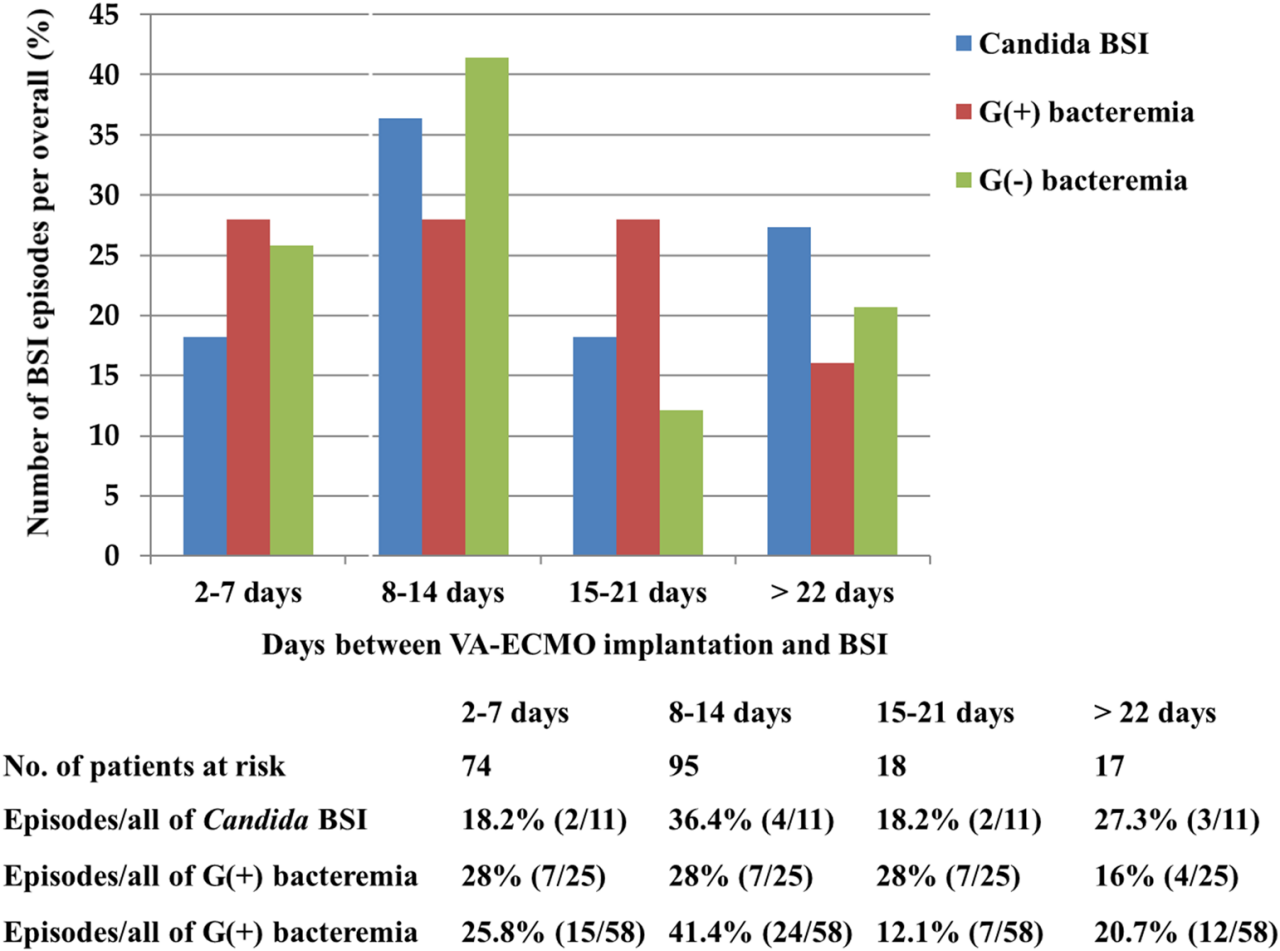
Number of each type of BSI episodes to the overall number of BSI episodes (%) among VA ECMO patients that was stratified by days between ECMO implantation and BSI.

**Figure 2:**
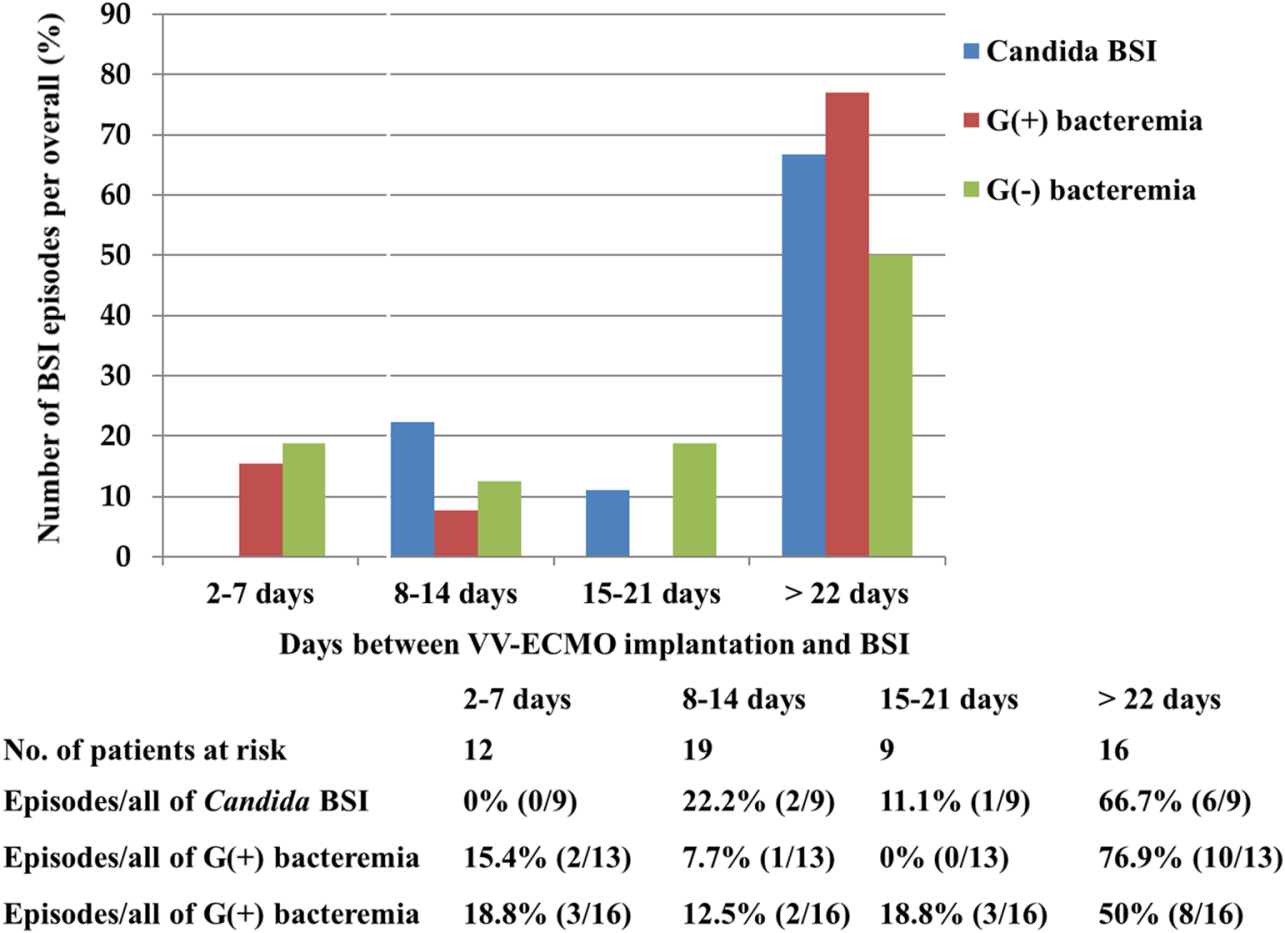
Number of each type of BSI episodes to the overall number of BSI episodes (%) among VV ECMO patients that was stratified by days between ECMO implantation and BSI.

In the first 2 weeks after VA ECMO implantation, 59 (62.8%) of the 94 BSI episodes of BSI occurred (Fig. 1). Of the 59 episodes, gram-negative bacteremia (39/59, 66.1%) were the most commonly identified BSI type; 39/58 (67.2%) of gram-negative bacteremia, 14/25 (56%) of gram-positive bacteremia, and 6/11 (54.5%) of *Candida* BSI episodes were identified. After 3 weeks of VV ECMO implantation, 24/38 (63.2%) BSI episodes occurred (Fig. 2). More than half of each type of BSIs (8/16, 50% of gram-negative bacteremia; 10/13, 76.9% of gram-positive bacteremia; 6/9, 66.7% of *Candida* BSI) were detected under VV ECMO after 3 weeks of VV ECMO implantation. The prevalence of BSIs during ECMO in VA ECMO and VV ECMO at 33.0% (64/194) and 30.4% (17/56) was the highest in gram-negative bacteremia (50/194, 25.8% vs. 12/56, 21.4%) and the lowest in *Candida* BSI (8/194, 4.1% vs. 9/56, 16.1%), respectively. The incidence of BSIs during ECMO in VA ECMO and VV ECMO was 44.26 vs. 16.56 episodes/1,000 ECMO days, respectively. Gram-negative bacteremia had the highest incidence both in VA ECMO and VV ECMO (51.33 vs. 19.07 episodes/1,000 ECMO days). *Candida* BSI incidence, which was the lowest in VA ECMO (33.43 episodes/1,000 ECMO days), was higher than that of gram-positive bacteremia (16.48 episodes/1,000 ECMO days vs. 14.30 episodes/1,000 ECMO days) in VV ECMO.

### Comparison of characteristics and outcomes of Candida BSI and bacteremia

Tables 3 and 4 lists the differences in clinical features and outcomes between *Candida* BSI and bacteremia during VA and VV ECMO.

**Table 3:** Clinical characteristics and outcomes of the 11 cases of *Candida* BSI and 83 cases of bacteremia during venoarterial extracorporeal membrane oxygenation (VA ECMO).

Variables	*Candida* BSI (n = 11)	Bacteremia		*P* value* (*Candida* BSI vs. Gram- negative bacteremia	*P* value* (*Candida* BSI vs. Gram-positive bacteremia
		Gram-negative bacteremia (n = 58)	Gram-positive bacteremia (n = 25)		
**Age, median years (IQR)**	63 (54–68)	56 (45–68)	59 (45–66)	0.44	0.46
Male	5 (46)	44 (76)	20 (80)	0.042	0.038
**ICU days before ECMO implantation, median (IQR)**	1 (1–2)	0 (0–2)	0 (0–2)	0.11	0.13
**Time between ECMO implantation and BSI, median days (IQR)**	14 (8–35)	12 (7–18)	13 (6–19)	0.29	0.48
**Duration of ECMO, median days (IQR)**	23 (14–42)	17 (12–23)	20 (15–30)	0.08	0.50
**ECPR**	1 (9)	13 (22)	10 (40)	0.44	0.12
**Immunocompromised state**	4 (36)	34 (59)	17 (68)	0.20	0.14
Hematological malignancy	0	1 (2)	1 (4)	> 0.99	> 0.99
Active solid tumor	0	1 (2)	0	> 0.99	N/A
Solid-organ transplant	1 (9)	2 (3)	2 (8)	0.41	> 0.99
Long-term corticosteroid use	0	1 (2)	0	> 0.99	N/A
High-dose corticosteroid use	4 (36)	31 (53)	17 (68)	0.34	0.14
**SAPS III at ICU admission**	57 (44–64)	53 (37–65)	49 (42–60)	0.39	0.42
**SOFA at the time of BSI**	12 (8–15)	13 (11–14)	11 (9–13)	0.60	0.69
**SAPS II at the time of BSI**	53 (49–67)	64 (53–81)	62 (51–73)	0.17	0.64
**Inflammatory markers on the day of BSI**					
Lactate, mmol/L	3.5 (2.9–13.7)	5.0 (2.9–9.8)	4.3 (2.5–5.6)	0.57	0.89
CRP, mg/dL	11.4 (6.4–25.0)	13.9 (5.4–30.0)	13.0 (8.0–24.1)	0.41	0.66
Procalcitonin, ng/mL	2.4 (1.5–7.9)	5.0 (1.3–19.3)	5.5 (1.8–15.9)	0.23	0.29
**CRRT prior to BSI**	10 (91)	44 (76)	19 (76)	0.43	0.40
TPN prior to BSI	8 (73)	30 (52)	18 (72)	0.32	> 0.99
**Total antibiotic treatment duration before BSI, median days (IQR)**^a^	20 (9–41)	14 (8–27)	13 (9–23)	0.21	0.31
**Antibiotic exposure prior to BSI**^b^					
Glycopeptide	8 (73)	34 (59)	14 (56)	0.51	0.47
Carbapenem	9 (82)	20 (35)	10 (40)	**0.006**	**0.03**
Piperacillin/tazobactam	3 (27)	31 (53)	13 (52)	0.19	0.28
Amikacin	2 (18)	5 (9)	5 (20)	0.31	> 0.99
Fluoroquinolones	6 (55)	17 (29)	8 (32)	0.10	0.20
Colistin	7 (64)	20 (35)	12 (48)	0.10	0.48
Metronidazole	2 (18)	11 (19)	2 (8)	> 0.99	0.57
Antifungal agent	1 (9)	6 (10	2 (8)	> 0.99	> 0.99
**Outcomes**					
ECMO weaning	2 (18)	18 (31)	6 (24)	0.49	> 0.99
Survival to discharge^c^	1/3 (33)	8/48 (17)	4/13 (31)	0.43	0.68

BSI, bloodstream infection; CRP, C-reactive protein; CRRT, continuous renal replacement therapy; ECPR, extracorporeal cardiopulmonary resuscitation; ICU, intensive care unit; IQR, interquartile range; SAPS, Simplified Acute Physiology Score; SOFA, sequential organ failure assessment; TPN, total parenteral nutrition.

*P values with significant difference are indicated in **bold.**

Data are presented as No. (%) unless indicated otherwise.

^a^Total antibiotic treatment duration between hospital admission and BSI development.

^b^ > 48 h of therapy within the 14 days prior to the BSI.

^c^ > Overall survival rates evaluated in patient-based, not episode-based.

**Table 4:** Clinical characteristics and outcomes of the 9 cases of *Candida* BSI and 29 cases of bacteremia during venovenous extracorporeal membrane oxygenation (VV ECMO).

Variables	*Candida* BSI (n = 9)	Bacteremia		*P* value (*Candida* BSI vs. Gram- negative bacteremia	*P* value (*Candida* BSI vs. Gram- positive bacteremia
		Gram-negative bacteremia (n = 16)	Gram-positive bacteremia (n = 13)		
**Age, median years (IQR)**	56 (46–63)	57 (40–68)	57 (53–64)	0.72	0.60
**Male**	6 (67)	11 (69)	9 (69)	> 0.99	> 0.99
**ICU days before ECMO implantation, median (IQR)**	3 (1–23)	4 (1–10)	2 (0–14)	> 0.99	0.56
**Time between ECMO implantation and BSI, median days (IQR)**	30 (17–66)	23 (9–40)	28 (18–80)	0.39	0.79
**Duration of ECMO, median days (IQR)**	49 (25–92)	43 (24–51)	49 (35–107)	0.52	0.70
**Immunocompromised state**	7 (78)	10 (63)	13 (100)	0.66	0.16
Hematological malignancy	0	0	0	N/A	N/A
Active solid tumor	1 (11)	0	0	0.36	0.41
Solid-organ transplant	2 (22)	4 (25)	5 (39)	> 0.99	0.65
Long-term corticosteroid use	1 (11)	0	1 (8)	0.36	> 0.99
High-dose corticosteroid use	5 (56)	6 (38)	10 (77)	0.43	0.38
**SAPS III at ICU admission**	56 (28–62)	40 (21–53)	42 (38–64)	0.21	0.97
**SOFA at the time of BSI**	11 (6–14)	11 (7–16)	9 (8–11)	0.72	0.60
**SAPS II at the time of BSI**	49 (28–54)	49 (37–57)	46 (38–54)	0.89	0.95
**Inflammatory markers on the day of BSI**					
Lactate, mmol/L	2.8 (2.1–3.5)	3.3 (2.7–5.3)	3.1 (2.3–4.3)	0.23	0.74
CRP, mg/dL	14.7 (7.1–21.5)	15.6 (8.2–21.4)	21.0 (11.6–28.5)	0.72	0.21
Procalcitonin, ng/mL	1.3 (0.6–4.1)	2.2 (1.2–7.4)	2.2 (1.2–7.4)	0.30	0.95
**CRRT prior to BSI**	6 (67)	13 (81)	10 (77)	0.63	0.66
**TPN prior to BSI**	7 (78)	10 (63)	8 (62)	0.66	0.65
**Total antibiotic treatment duration before BSI, median days (IQR)**	35 (30–88)	35 (18–54)	41 (31–81)	0.42	0.95
**Antibiotic exposure prior to BSI**^a^					
Glycopeptide	8 (89)	14 (88)	9 (69)	> 0.99	0.36
Carbapenem	3 (33)	10 (63)	5 (39)	0.23	> 0.99
Piperacillin/tazobactam	5 (56)	9 (56)	5 (39)	> 0.99	0.67
Amikacin	1 (11)	1 (6)	2 (15)	> 0.99	> 0.99
Fluoroquinolones	3 (33)	4 (25)	2 (15)	0.68	0.61
Colistin	5 (56)	11 (69)	9 (69)	0.67	0.66
Metronidazole	0	0	0	N/A	N/A
Antifungal agent	2 (22)	7 (44)	6 (46)	0.40	0.38
**Outcomes**					
ECMO weaning	3 (33)	5 (31)	3 (23)	> 0.99	0.66
Survival to discharge^b^	2/9 (22)	1/5 (20)	2/4 (50)	> 0.99	0.53

BSI, bloodstream infection; CRP, C-reactive protein; CRRT, continuous renal replacement therapy; ICU, intensive care unit; IQR, interquartile range; SAPS, Simplified Acute Physiology Score; SOFA, sequential organ failure assessment; TPN, total parenteral nutrition.

Data are presented as No. (%) unless indicated otherwise.

^a^ > 48 h of therapy within the 14 days prior to the BSI.

^b^ > Overall survival rates evaluated in patient-based, not episode-based.

In VA ECMO, there were no significant differences in clinical features, including the timing of BSI development, severity scores, and several important inflammatory markers, between *Candida* BSI and bacteremia. However, the duration of ECMO in episodes of *Candida* BSI was longer than that in episodes of gram-negative bacteremia despite the lack of significance (median [IQR]; 23 [14–42] vs. 17 [12–23] days; *P* = 0.08). In addition, prior exposure to carbapenem was independently associated with *Candida* BSI; this was not the case for gram-negative (9/11, 82% vs. 20/58, 35%, *P* = 0.006) and gram-positive bacteremia (9/11, 82% vs. 10/25, 40%, *P* = 0.03). In VV ECMO, there were no significant differences in the clinical features, including timing of the development of BSI, severity scores, several important inflammatory markers, and prior antibiotic exposure, between *Candida* BSI and bacteremia. There were also no significant differences in outcomes such as ECMO weaning and survival to discharge between *Candida* BSI and bacteremia.

## Discussion

In the present study, we evaluated whether there were differences in the clinical characteristics and outcomes of BSI under VA and VV ECMO and whether these differ between *Candida* BSI and bacteremia in adult ECMO patients. The results indicate that the types and clinical features of BSI were distinct in each mode of BSI; early-onset gram-negative bacteremia after ECMO implantation with high levels of severity scores and inflammatory markers were more prominent in VA ECMO, while late-onset *Candida* BSI after ECMO implantation was more common in VV ECMO. Interestingly, *Candida* BSI was not uncommon in the first 2 weeks after VA ECMO implantation, while most *Candida* BSIs occurred after 3 weeks of VV ECMO implantation. Furthermore, our results suggested no specific differences in the clinical characteristics and outcomes between *Candida* BSI and bacteremia during VA and VV ECMO, except that *Candida* BSI was significantly associated with prior exposure to carbapenem; this was not the case for gram-negative and gram-positive bacteremia in VA ECMO.

The infectious agents involved in VA and VV ECMO may be different because the main indications of these procedures differ. Patients requiring VV ECMO are commonly those with respiratory failure such as acute respiratory distress syndrome and presumed primary lung infection, while patients requiring VA ECMO have heterogenous conditions resulting in cardiogenic shock with multi-organ failure^23,24^. We posit that the underlying morbidity may influence the epidemiology of infectious diseases in VA and VV ECMO; thus, the clinical characteristics and outcomes of BSI may differ from each other depending on the modes of ECMO. Our study is valuable because of the scarcity of comparative studies that have specifically evaluated the differences in characteristics of BSI during VA and VV ECMO. Moreover, our study has strength because of its larger number of BSI episodes in adult ECMO patients than that in previous studies^2–8,12–15^. It is noteworthy that the timing of BSI as well as infectious agents may differ in VA and VV ECMO, which highlights the importance of appropriate empirical antibiotic use particularly in the absence of typical signs of sepsis in ECMO patients^25^.

Important findings in the present study were the characteristics of *Candida* BSI in adult patients receiving VA and VV ECMO. Although there have been a few reports on fungal infections in adult ECMO patients^11,26,27^, no comparative studies that specifically evaluated the differences in characteristics of *Candida* BSIs depending on the mode of ECMO and that made a comparison with bacteremia were available. *Candida* BSI occurrence was not uncommon in patients under VA ECMO within 2 weeks after ECMO implantation and was associated with prior carbapenem exposure in our study. Therefore, empiric antifungal therapy in the early course of VA ECMO might be warranted in hemodynamically unstable patients receiving carbapenem. In contrast, *Candida* BSI was more common in VV ECMO than in VA ECMO and two thirds of them occurred after 3 weeks of ECMO implantation. This indicated that empiric antifungal therapy might be warranted in patients with prolonged ECMO therapy. Our result may be reinforced by the reports of previous studies showing increased mortality following fungal infections in adult ECMO patients^3,11^ and lower rates of fungal infections with routine fluconazole prophylaxis in pediatric patients^28^. The currently updated Infectious Diseases Society of America (IDSA) guidelines suggest the use of antifungal agents for high-risk adult patients in ICUs with a high incidence (5%) of invasive candidiasis^29^. We believe that it would be worthwhile to evaluate the effect of antifungal prophylaxis or empiric antifungal therapy on outcomes in targeted ECMO patients, particularly when a high incidence of *Candida* BSI is expected, such as within 2 weeks of VA ECMO implantation or after 3 weeks of VV ECMO implantation, as reported in our study.

Notably, there were no distinct differences in clinical features, such as the timing of BSI, underlying immunosuppression, severity scores, and levels of inflammatory markers, between *Candida* BSI and bacteremia both in VA and VV ECMO. The unreliability of signs to predict the specific type of BSI may suggest the usefulness of the blood culture surveillance for ECMO patients. However, previous studies regarding the usefulness of routine blood culture surveillance were mostly performed for pediatric ECMO patients^30–33^, and the role of routine blood culture surveillance in ECMO patients remains inconclusive^34,35^. Our results suggested that BSI occurred frequently in the early phase of the first 2 weeks after VA ECMO implantation (59/94, 62.8%) and in the late phase of more than 3 weeks after VV ECMO implantation (24/38, 63.2%), which indicated that routine blood culture surveillance might be warranted for adult ECMO patients, particularly during the period of ECMO therapy. In addition, we believe that the present study has important clinical implications for future research evaluating whether the concurrent use of rapid non-culture-based diagnostics with blood culture surveillance may help improve the clinical practice of care for *Candida* BSI and bacteremia in adult ECMO patients^36,37^.

The prevalence and incidence of BSI in the present study were higher than those in a previous study in adult patients^1^. The possible difficulty in detecting classical findings of sepsis in BSI patients during ECMO therapy influenced daily blood culture surveillance practices in our center^33,35^. However, there are limited data on these values in heterogenous adult patient populations, and the practice of daily blood culture surveillance, antibiotic prophylaxis regimen, and infection prevention measures, varied widely in these studies^2,4–6,12–14,27,35^. A well-organized large sample-sized study to evaluate the recommendations for BSI prevention and daily surveillance blood cultures during VA and VV ECMO is needed to reduce the controversy about the true prevalence and incidence of BSI. In addition, specific prevention practices, such as chlorhexidine bathing, should be verified further to decrease the prevalence and incidence of BSI during VA and VV ECMO^38^.

This study has several limitations. First, this was a single center study; therefore, there can be other important factors including ECPR and site of ECMO cannulation that could have caused variations in results of other institutions. Thus, this precludes external generalizability. Second, the retrospective nature of our present analyses may have resulted in several biases or missing data that could have influenced the results. Third, a small number of patients without multivariable analysis could lead to an underpowering of the results. Fourth, we did not analyze the differences in clinical characteristics such as value of inflammatory markers and severity of illness between BSI and non-BSI patients. Despite several limitations, our study provides valuable findings, given the absence of comparative studies that examined the differences in BSIs between VA and VV ECMO and between *Candida* BSI and bacteremia. Prospective, multicenter studies should be performed to evaluate the epidemiology and meaningful predictors of BSI in adult patients receiving VA and VV ECMO, which can help physicians implement targeted clinical practice according to the mode and timing of ECMO therapy. In addition, based on the information of comparison between *Candida* BSI and bacteremia, future studies with large scale may help to clarify the role of antifungal prophylaxis and daily surveillance blood cultures in the absence of typical signs of sepsis in ECMO patients.

## Conclusions

The mode of ECMO may affect the clinical features and timing of the specific type of BSI. In particular, *Candida* BSI occurs not uncommonly during the first 2 weeks of the VA ECMO course, especially in patients with prior exposure to carbapenem, while it usually occurs during the late course of more than 3 weeks after VV ECMO implantation. Consequently, routine blood culture surveillance and empiric antifungal therapy might be warranted in targeted adult ECMO patient populations, regardless of the levels of inflammatory markers and severity scores of the patients.

### Ethics approval and consent to participate

The study was approved by the Hallym University Sacred Heart Hospital Institutional Review Board (IRB), which waived the requirement for informed consent due to the retrospective design of the study.

## Data availability statements

The datasets generated during and/or analyzed during the current study are available from the corresponding author on reasonable request.

## Abbreviations

BSI: Bloodstream infection
CRRT: Continuous renal replacement therapy
CRP: C-reactive protein
ECMO: Extracorporeal membrane oxygenation
ICU: Intensive care unit
IDSA: Infectious Diseases Society of America
IQR: Interquartile range
SAPS: Simplified acute physiology score
SOFA: Sequential organ failure assessment
TPN: Total parenteral nutrition
VA: Venoarterial
VV: Venovenous
